# Serum Homocysteine Concentration Is Significantly Associated with Inflammatory/Immune Factors

**DOI:** 10.1371/journal.pone.0138099

**Published:** 2015-09-14

**Authors:** Tianyu Li, Yang Chen, Jie Li, Xiaobo Yang, Haiying Zhang, Xue Qin, Yanling Hu, Zengnan Mo

**Affiliations:** 1 Center for Genomic and Personalized Medicine, Guangxi Medical University, Nanning, Guangxi Zhuang Autonomous Region, China; 2 Medical Scientific Research Centre, Guangxi Medical University, Nanning, Guangxi Zhuang Autonomous Region, China; 3 Institute of Urology and Nephrology, First Affiliated Hospital of Guangxi Medical University, Nanning, Guangxi Zhuang Autonomous Region, China; 4 Department of occupational health and environmental health, School of public health of Guangxi Medical University, Nanning, Guangxi Zhuang Autonomous Region, China; 5 Department of Clinical Laboratory, First Affiliated Hospital of Guangxi Medical University, Nanning, Guangxi Zhuang Autonomous Region, China; 6 Guangxi key laboratory for genomic and personalized medicine, Guangxi collaborative innovation center for genomic and personalized medicine, Nanning, Guangxi Zhuang Autonomous Region, China; 7 Research Center for Guangxi Reproductive Medicine, First Affiliated Hospital of Guangxi Medical University, Nanning, Guangxi Zhuang Autonomous Region, China; CSIR-INSTITUTE OF GENOMICS AND INTEGRATIVE BIOLOGY, INDIA

## Abstract

Recent studies suggest that serum homocysteine (HCY) level is correlated to inflammatory/immune factors that influence the development and progression of many diseases, such as cardiovascular disease. However, the association between serum HCY level and inflammatory/immune factors in healthy populations has not been systematically investigated. This study was conducted based on the Fangchenggang Area Male Health and Examination Survey (*FAMHES*) project. After comprehensive baseline analysis, we could not find any significant association between HCY level and inflammatory/immune factors. However, in the next linear regression analysis, serum C4 [age-adjusted: *Beta* = -0.053, 95%*CI* = (-3.798, -0.050), *P* = 0.044; multivariate adjusted: *Beta* = -0.064, 95%*CI* = (-4.271, -0.378), *P* = 0.019] and C-reactive protein (CRP) concentration [unadjusted: *Beta* = 0.056, 95%*CI* = (0.037, 0.740), *P* = 0.030] were positively related with HCY. In further binary regression analysis, a significant correlation was confirmed for C4 and HCY [age-adjusted: *OR* = 0.572, 95%*CI* = (0.359, 0.911); multivariate adjusted: *OR* = 0.558, 95%*CI* = (0.344, 0.905)]. In order to discover more potential associations, multivariate logistic regression analysis was applied and suggested that HCY and C4 were significantly correlated [age-adjusted: *OR* = 0.703, 95%*CI* = (0.519, 0.951); multivariate adjusted: *OR* = 0.696, 95%*CI* = (0.509, 0.951)]. In addition, immunoglobulin M (IgM) may influence the HCY level to some extent [unadjusted: *OR* = 1.427, 95%*CI* = (1.052, 1.936); age-adjusted: *OR* = 1.446, 95%*CI* = (1.061, 1.970); multivariate adjusted: *OR* = 1.447, 95%*CI* = (1.062, 1.973)]. Combining our results with recent studies, we propose that C4, CRP, and IgM in serum are significantly associated with HCY concentration. Further studies are needed on the mechanism of the interaction, especially among cardiovascular disease subjects.

## Introduction

Homocysteine (HCY) is an amino acid that is produced during the metabolism of methionine, in which many coenzymes and co-factors are involved [1]. In this process, two important intermediates are generated: S-adenosylmethionine (*SAM*) and S-adenosylhomocysteine (*SAH*) [2]. Generally, the ratio of *SAM* to *SAH* should be balanced [3]. Under certain circumstances, this balance is disrupted and the imbalance may be associated with certain diseases such as inflammatory bowel diseases, type 2 diabetes, coronary artery disease, stroke, etc [4–7]. The serum HCY concentrations were treated as an independent risk marker, especially for cardiovascular diseases [8–9]. It has been proposed that an elevated HCY level in blood could induce endothelial dysfunction, increase vascular production of reactive oxygen species (*ROS*), and decrease the bioavailability of endothelial nitric oxide (*NO*), which may be regarded as the precursor lesions of atherosclerosis [10–13].

Additionally, activating the immune system might also play a key role in the development and progression of cardiovascular diseases. In this process, many immune cells and inflammatory factors—such as macrophages, dendritic cells, T lymphocytes, B lymphocytes, complement proteins, interleukins, tumor necrosis factor, etc.—are activated and secreted [14–19]. Additionally, some other inflammatory/immune elements, including C-reactive protein (CRP), adhesion molecules, and metalloproteinases, have also been confirmed in recent studies to be positively associated with the level of HCY [20–22]. However, the association between serum HCY level and inflammatory/immune factors in healthy populations has not been systematically investigated.

In order to clarify the association between HCY concentrations and inflammatory/immune factors, this cross-sectional study was conducted on the basis of the existing data of our Fangchenggang Area Male Health and Examination Survey (*FAMHES*) project. This study was designed to investigate the potential association between CRP, complement components, immunoglobulins, and HCY level among healthy individuals, and it was envisaged that it might provide additional markers for HCY-related diseases and help us understand their development and progression on the level of inflammatory/immune regulation.

## Methods and Materials

### Participants

This population-based study was based on the *FAMHES* project. The details of the participants have been described in another study [23]. Briefly, this project was focused on the relationships between environmental and genetic factors, and involved 4303 non-institutionalized Chinese men aged 17 to 88 years old in the Fangchenggang Area of Guangxi. The participants all took part in a routine physical examination at the Medical Center in Fangchenggang First People’s Hospital from September to December 2009. After a comprehensive demographic and health survey, data from 3593 participants in the form of interviews were collected. The response rate was 83.5% [24]. Written informed consent was obtained for all participants and their guardians. In addition, we removed juvenile subjects and ensured that only data from adult participants (age ≥18 years) were included in this analysis. There were no significant differences between the men who participated in the interviews and those who did not. The study was approved by the medical ethics committee of Guangxi Medical University.

### Sample selection

This study investigated the association between HCY level in blood and inflammatory/immune parameters based on available data from the *FAMHES* project. In this analysis, six available inflammatory/immune factors were included [complement 3 (C3), complement 4 (C4), high-sensitivity C-reactive protein (CRP), immunoglobulin A (IgA), immunoglobulin G (IgG), and immunoglobulin M (IgM)]. Every item was treated as an independent factor to explore the correlations between these items and HCY level.

In the process of screening participants, we defined the exclusion criteria as follows: (I) without complete values of inflammatory/immune parameters (C3, C4, CRP, IgA, IgG, and IgM) and HCY levels; (II) without complete individual information of participants; (III) currently with hypertension, myocardial infarction, congestive heart failure, stroke, hyperthyroidism, rheumatoid arthritis, acquired immune deficiency syndrome, any kind of cancer, or having a history of trauma/surgery/inflammation; (IV) taking medicines that could influence inflammation/immunity, such as non-steroidal anti-inflammatory drugs, antibiotics, cimetidine, glucocorticoids, or other steroidal drugs; (V) inflammatory/immune items in our analysis were not in the normal ranges according to laboratory standards (normal ranges: C3 0.8–1.5 g/L, and C4 0.2–0.6 g/L, CRP 0–10 mg/L, IgA 0.7–3.5 g/L, IgM 0.5–2.6 g/L, IgG 7.0–16.6 g/L), which was identified as acute infection, inflammatory reaction, or as being immunocompromised.

### Phenotypes and covariates

As the main object of this study, the values of HCY level were collected. According to the threshold, the normal value of HCY level was restricted to 5–15 μmol/L. When the values exceeded 15 μmol/L, hyperhomocysteinemia (HHCY) was defined [25]. After screening our participants according to the above criteria, 1339 males were included in the analysis for complement C3 (780 subjects with normal HCY level, and 559 with HHCY). There were 1436 participants included in the analysis for complement C4 (840 subjects with normal HCY level, and 596 with HHCY). As for CRP, 1471 participants were involved (862 subjects with normal HCY level, and 609 with HHCY). For IgA, 1336 eligible participants were included in the analysis (786 subjects with normal HCY level, and 550 with HHCY). Meanwhile, 1384 samples were studied for the IgG (821 subjects with normal HCY level, and 563 with HHCY). In addition, 1387 sets of data for IgM were included (820 subjects with normal HCY level, and 567 with HHCY).

In addition to the main items in the analysis, we also applied a comprehensive questionnaire in the survey during a face-to-face interview. The essential characteristics (age, sex, smoking, drinking, etc.) and physical examinations (height, weight, waistline, hipline, etc.) of all the males were collected. The interviews were conducted by trained personnel using a standardized protocol. In this study, smoking status was classified as never a smoker or currently a smoker. Alcohol consumption was defined as the consumption of alcohol, including beer, wine, and hard liquor, within the participant’s lifetime. For the physical examination, body weight with thin clothing and height without shoes were measured, and then body mass index (BMI) was calculated. Additionally, waist circumference was measured at the midpoint between the inferior costal margin and the superior iliac crest on the mid-axillary line. The hipline was defined as the maximum circumference over the buttocks. After transformation, the waist-hip ratio (WHR) was categorized as normal weight (WHR ≤ 0.9) and obese (WHR > 0.9) [26].

### Serum measurements

Blood samples were collected from participants between 8:00 and 11:00 a.m. after they had fasted for at least 8 h (overnight). All the samples were transported to the testing center of the Department of Clinical Laboratory at the First Affiliated Hospital of Guangxi Medical University in Nanning within 2–3 h. They were centrifuged within 15–25 min, and stored at −80°C. The *C3*, *C4*, *CRP*, *IgA*, *IgM*, and *IgG* levels were detected with electrochemiluminescence immunoassays and immunoturbidimetric methods. All details were presented in our previous publications [23–24, 27].

### Statistical analysis

In our first analysis, all the continuous variables were tested for Gaussian distribution by the method of the Shapiro—Wilks test. In order to ensure the data had approximately Gaussian distribution, the items (C3, C4, CRP, IgA, IgG, and IgM) were logarithmically transformed in the subsequent analysis. Then, on the basis of a threshold of 15 μmol/L, HCY concentrations were categorized into normal and higher groups. The Student's t test, Mann—Whitney U test, and the Χ^2^ test were applied where appropriate.

After evaluating the potential confounding factors and then the association between HCY level and inflammatory/immune parameters after adjusting for these covariates, the regression analyses were conducted. There were three adjusted groups (unadjusted, age-adjusted, and multivariate-adjusted models) that were used in both linear regression and binary logistic regression. In the multivariate-adjusted models, the following covariates were applied: age, smoking status, alcohol consumption, BMI, and WHR. In addition, in order to confirm the results in the anterior analyses and to discover more potential associations, we applied multivariate logistic regression analysis. Each inflammatory/immune parameter (C3, C4, CRP, IgA, IgG, and IgM) was divided into quartiles according to the values of serum concentrations. Then, an associated analysis was conducted between HCY levels and each group of inflammatory/immune items with the same three adjusted schemes above. All analyses were performed with SPSS version 16.0 software (SPSS Inc., Chicago, IL, USA). The statistical tests were two-tailed.

## Results

### Features of the baseline analysis

In the baseline analysis, there was no significant relationship between any inflammatory/immune parameters (C3: *P* = 0.763; C4: *P* = 0.086; CRP: *P* = 0.356; IgA: *P* = 0.940; IgG: *P* = 0.509; IgM: *P* = 0.332). As for the other characteristic factors, there was a positive relationship between WHR and IgM (C3: *P* = 0.072; C4: *P* = 0.142; CRP: *P* = 0.053; IgA: *P* = 0.055; IgG: *P* = 0.054; IgM: *P* = 0.042). However, the same potential correlations were not identified with *BMI* (C3: *P* = 0.235; C4: *P* = 0.627; CRP: *P* = 0.277; IgA: *P* = 0.076; IgG: *P* = 0.113; IgM: *P* = 0.524), smoking status (C3: *P* = 0.602; C4: *P* = 0.757; CRP: *P* = 0.400; IgA: *P* = 0.337; IgG: *P* = 0.592; IgM: *P* = 0.285), or alcohol consumption (C3: *P* = 0.200; C4: *P* = 0.545; CRP: *P* = 0.430; IgA: *P* = 0.571; IgG: *P* = 0.584; IgM: *P* = 0.633).

### Inflammatory/immune parameters associated with HCY level

In order to investigate the association between inflammatory/immune parameters and HCY concentration, linear regression and binary logistic regression analyses were conducted. In these analyses, three adjusted groups were applied, in which the covariates were age, smoking status, alcohol consumption, BMI, and WHR. The results of the linear regression analysis indicate that C4 level in blood might be significantly associated with HCY after adjusting for age and other factors [unadjusted: *Beta* = -0.045, 95%*CI* = (-3.499, 0.232), *P* = 0.232; age-adjusted: *Beta* = -0.053, 95%*CI* = (-3.798, -0.050), *P* = 0.044; multivariate adjusted: *Beta* = -0.064, 95%*CI* = (-4.271, -0.378), *P* = 0.019].

For the CRP, a positive relationship with the HCY level was also identified [unadjusted: Beta = 0.056, 95%CI = (0.037, 0.740), *P* = 0.030; age-adjusted: *Beta* = 0.046, 95%*CI* = (-0.039, 0.673), *P* = 0.081; multivariate adjusted: *Beta* = 0.043, 95%*CI* = (-0.085, 0.678), *P* = 0.127]. In the binary logistic regression analysis, inflammatory/immune parameters were related to HCY level, especially for C4 [unadjusted: *OR* = 0.672, 95%*CI* = (0.426, 1.059), *P* = 0.087; age-adjusted: *OR* = 0.572, 95%*CI* = (0.359, 0.911), *P* = 0.019; multivariate adjusted: *OR* = 0.558, 95%*CI* = (0.344, 0.905), *P* = 0.018]. However, there were no other correlations on the basis of these analyses. These results and baseline features are shown in Tables 1–4.

**Table 1:** The baseline features of samples by dividing into two groups (normal and hyperhomocysteinemia) to describe the association between complement 4 (C4) and homocysteine (HCY).

	Total	normal (5–15μmol/L)	HHCY (>15μmol/L)	*P* value
Number	1436	840	596	
Age		35.14±9.59	38.17±11.98	**<0.001**
C4		0.34±0.08	0.33±0.08	0.086
BMI (kg/m^2^)		23.20±3.43	23.29±3.20	0.627
WHR (%)				
≤0.9		549	367	
>0.9		291	229	0.142
Smoking (%)				
No		368	266	
Yes		472	330	0.757
Drinking (%)				
No		120	92	
Yes		720	504	0.545

The threshold value of homocysteine was defined as 15μmol/L.

**Table 2:** The baseline features of samples by dividing into two groups (normal and hyperhomocysteinemia) to describe the association between C reactive protein (CRP) and homocysteine (HCY).

	Total	normal (5–15μmol/L)	HHCY (>15μmol/L)	*P* value
Number	1471	862	609	
Age		35.07±9.54	38.20±11.98	**<0.001**
CRP		0.98±0.05	1.05±1.41	0.356
BMI (kg/m^2^)		23.20±3.42	23.39±3.22	0.277
WHR (%)				
≤0.9		569	372	
>0.9		293	237	0.053
Smoking (%)				
No		373	277	
Yes		489	332	0.400
Drinking (%)				
No		119	93	
Yes		743	516	0.430

The threshold value of homocysteine was defined as 15μmol/L.

**Table 3:** Association between inflammation/immune substances and HCY level in the regression analysis.

HCY level																		
		C3			C4			CRP			IgA			IgG			*IgM*	
	*Beta*	95%*CI*	*P*	*Beta*	95%*CI*	*P*	*Beta*	95%*CI*	*P*	*Beta*	95%*CI*	*P*	*Beta*	95%*CI*	*P*	*Beta*	95%*CI*	*P*
Unadjusted	-0.003	-3.214, 2.872	0.912	-0.045	-3.499, 0.232	0.086	**0.056**	**0.037, 0.740**	**0.030**	0.031	-0.642, 2.339	0.264	-0.009	-3.238, 2.275	0.732	-0.027	-1.711, 0.544	0.310
Age-adjusted	-0.07	-3.422, 2.670	0.809	**-0.053**	**-3.798, -0.050**	**0.044**	0.046	-0.039, 0.673	0.081	0.027	-0.737, 2.242	0.322	-0.201	-3.038, 2.473	0.841	-0.023	-1.626, 0.629	0.386
Multivariate adjusted	-0.031	-5.143, 1.623	0.308	**-0.064**	**-4.271, -0.378**	**0.019**	0.043	-0.085, 0.678	0.127	0.025	-0.819, 2.199	0.370	-0.007	-3.196, 2.439	0.792	-0.024	-1.656, 0.623	0.374

Multivariate adjusted for age, smoking status, alcoholic drinking, *BMI*, *WHR*.

As a continuous variable, *HCY* levels were treated as dependent variable analyzed by linear regression.

*HCY* = homocysteine; *BMI* = Body Mass Index; *WHR* = waist hip rate; *C3* = Complement *C3*; *C4* = Complement *C4*; *CRP* = C reactive protein; *Ig* = Immunoglobulin.

**Table 4:** Association between inflammatory/immune substances and *HCY* in the regression analysis.

HHCY (dichotomy)																		
	C3			C4			CRP			IgA			IgG			*IgM*		
	*OR*	95%*CI*	*P*	*OR*	95%*CI*	*P*	*OR*	95%*CI*	*P*	*OR*	95%*CI*	*P*	*OR*	95%*CI*	*P*	*OR*	95%*CI*	*P*
Unadjusted	0.895	0.436, 1.836	0.762	0.672	0.426, 1.059	0.087	1.037	0.950, 1.131	0.417	1.014	0.712, 1.444	0.940	0.800	0.413, 1.550	0.509	1.145	0.871, 1.505	0.332
Age-adjusted	0.807	0.391, 1.666	0.562	**0.572**	**0.359, 0.911**	**0.019**	0.996	0.911, 1.089	0.931	0.968	0.678, 1.384	0.861	0.879	0.451, 1.715	0.706	1.206	0.915, 1.591	0.184
Multivariate adjusted	0.686	0.307, 1.536	0.360	**0.558**	**0.344, 0.905**	**0.018**	0.993	0.902, 1.092	0.878	0.943	0.657, 1.354	0.750	0.858	0.433, 1.699	0.660	1.204	0.910, 1.593	0.193

Multivariate adjusted for age, smoking status, alcoholic drinking, *BMI*, *WHR*.

As a binary variable, *HHCY* and normal *HCY* were treated as dependent variable analyzed by logistic regression.

*HCY* = homocysteine; *BMI* = Body Mass Index; *WHR* = waist hip rate; *C3* = Complement *C3*; *C4* = Complement *C4*; *CRP* = C reactive protein; *Ig* = Immunoglobulin.

In addition to the analyses above, we tried to confirm the significant association above and to investigate the new potential factors influencing the level of HCY. In this section, the multivariate logistic regression analysis was applied. The concentration in serum of each item (C3, C4, CRP, IgA, IgG, and IgM) collected in this study was divided into four-level scales by their levels (Q1<levels of 25%, 25%≤Q2≤50%, 50%<Q3≤75%, Q4>75%, showed in Table 5), which would be used in the associated analysis after adjusting for confounding factors. The results suggest that the high concentration of C4 (C4 level >75%) might be significantly associated with HCY [unadjusted: *OR* = 0.771, 95%*CI* = (0.572, 1.037); age-adjusted: *OR* = 0.703, 95%*CI* = (0.519, 0.951); multivariate adjusted: *OR* = 0.696, 95%*CI* = (0.509, 0.951)]. In addition, to our surprise, the *IgM* (25% ≤ IgM level ≤ 50%) was also related to the *HCY* level [unadjusted: *OR* = 1.427, 95%*CI* = (1.052, 1.936); age-adjusted: *OR* = 1.446, 95%*CI* = (1.061, 1.970); multivariate adjusted: *OR* = 1.447, 95%*CI* = (1.062, 1.973)] (Table 5).

**Table 5:** Results of multivariate logistic regression analysis for the association between *HCY* and six inflammatory/immune items.

	Quartile of items			
	Q1	Q2	Q3	Q4
HCY vs C3				
Model 1	1	1.017 (0.748, 1.382)	1.234 (0.908, 1.678)	0.917 (0.673, 1.249)
Model 2	1	1.021 (0.750, 1.390)	1.207 (0.886, 1.645)	0.868 (0.635, 1.186)
Model 3	1	0.983 (0.720, 1.341)	1.136 (0.823, 1.567)	0.808 (0.574, 1.137)
*HCY* vs *C4*				
Model 1	1	0.859 (0.639, 1.155)	0.980 (0.731, 1.314)	0.771 (0.572, 1.037)
Model 2	1	0.805 (0.596, 1.086)	0.887 (0.858, 1.196)	**0.703 (0.519, 0.951)**
Model 3	1	0.801 (0.592, 1.084)	0.874 (0.645, 1.185)	**0.696 (0.509, 0.951)**
HCY vs CRP				
Model 1	1	1.233 (0.919, 1.653)	1.043 (0.777, 1.401)	1.205 (0.899, 1.617)
Model 2	1	1.165 (0.866, 1.566)	0.901 (0.665, 1.220)	1.088 (0.807, 1.467)
Model 3	1	1.149 (0.850, 1.553)	0.877 (0.640, 1.203)	1.069 (0.776, 1.473)
HCY vs IgA				
Model 1	1	0.985 (0.733, 1.344)	0.935 (0.687, 1.273)	1.077 (0.792, 1.465)
Model 2	1	0.966 (0.706, 1.322)	0.911 (0.667, 1.243)	1.047 (0.768, 1.428)
Model 3	1	0.972 (0.711, 1.330)	0.899 (0.657, 1.228)	1.029 (0.752, 1.409)
HCY vs IgG				
Model 1	1	1.248 (0.923, 1.688)	0.917 (0.676, 1.244)	1.034 (0.763, 1.403)
Model 2	1	1.269 (0.935, 1.722)	0.967 (0.710, 1.316)	1.065 (0.783, 1.450)
Model 3	1	1.263 (0.930, 1.715)	0.959 (0.702, 1.309)	1.057 (0.773, 1.445)
HCY vs IgM				
Model 1	1	**1.427 (1.052, 1.936)**	1.248 (0.922, 1.690)	1.227 (0.904, 1.665)
Model 2	1	**1.446 (1.061, 1.970)**	1.304 (0.959, 1.772)	1.270 (0.932, 1.731)
Model 3	1	**1.447 (1.062, 1.973)**	1.294 (0.951, 1.760)	1.269 (0.929, 1.734)

The levels of six inflammatory/immune items were divided into quartile (Q1<levels of 25%, 25%≤Q2≤50%, 50%<Q3≤75%, Q4>75%).

Model 1: unadjusted; Model 2: adjust for age; Model 3: adjust for age, smoking status, alcoholic drinking, *BMI*, *WHR*.

HCY = homocysteine; BMI = Body Mass Index; WHR = waist hip rate; C3 = Complement C3; C4 = Complement C4; CRP = C reactive protein; Ig = Immunoglobulin.

## Discussion

HCY plays a key role in the development and progression of diseases, especially cardiovascular diseases [8–9]. It is widely believed that an elevated HCY level in blood can induce endothelial dysfunction, resulting in atherosclerosis and other cardiovascular diseases. In addition, the active inflammatory/immune systems might also take part in the lesion process [10–13]. In order to investigate the association between the related inflammatory/immune parameters and HCY concentration, this cross-sectional analysis was conducted based on the available items in our population-based *FAMHES* project. After comprehensive baseline, linear regression, binary logistic regression, and multivariate logistic regression analyses, our results can be summarized as follows: (I) age may be one of the significant confounding factors that can influence HCY levels; (II) C4 level especially for the high concentration was inversely correlated with HCY concentration; (III) IgM and CRP were also related to HCY level. Our study not only confirms the positive association between the HCY level and the inflammatory/immune system, but also paves the way for further research about the pathomechanisms of HCY-related diseases at the level of inflammatory/immune factors in the future.

In the baseline analysis, five essential items were included (age, BMI, WHR, smoking, and drinking). After comprehensive calculations, age was identified to be a significant factor for every inflammatory/immune parameter (C3, C4, CRP, IgA, IgG, and IgM). In the next regression analysis, the positive association between the C4 and HCY was discovered after adjusting for age. Based on these findings, we suggest that age may be one of the confounding factors that influence the level of HCY. In addition, WHR was also significantly associated with IgM (*P* = 0.042). In 2014, Bakulski et al. proposed that age might be correlated with HCY level [28]. Meanwhile, in 2010, Das et al. [29] conducted an analysis of HCY and lifestyle factors, such as smoking, alcohol intake, body weight, etc., which suggested that these factors potentially affect HCY level. Thus, on the basis of our results and other studies, the five items in our baseline analysis (age, BMI, WHR, smoking, and drinking) all might be key factors that influence the HCY concentration.

In order to investigate the association between inflammatory/immune parameters of the *FAMHES* project and the HCY level further, linear regression and binary logistic regression analyses were performed. Significant results were found for C4 and CRP. In the linear regression analysis, the values of HCY levels were treated as dependent factors. C4 was identified as influencing the HCY level only after adjusting for age or other confounding factors [unadjusted: *Beta* = -0.045, 95%*CI* = (-3.499, 0.232), *P* = 0.232; age-adjusted: *Beta* = -0.053, 95%*CI* = (-3.798, -0.050), *P* = 0.044; multivariate adjusted: *Beta* = -0.064, 95%*CI* = (-4.271, -0.378), *P* = 0.019]. Meanwhile, the HCY levels were divided into two-category data (a threshold of 15 μmol/L), and a binary logistic analysis was conducted. In this section, a positive correlation was also presented for C4 and HCY levels. The results suggest that C4 in the blood might increase inversely with HCY concentration. As a part of innate immune system, complement system is important in preventing bacterial infection, bridging the innate and adaptive immunity, and dealing with immune complexes, etc. Many proteins are involved in the process of activation of the complement system, in which C4 is a key part of the classical pathway and the lectin pathway [30]. Recently, C4 was identified to be associated with many diseases. In 2007, Giasuddin et al. [31] proposed that serum C4 might be profoundly elevated in acute myocardial infarction. Mosca et al. [32] suggested that an elevated C4 level could be associated with intermittent atopic asthma. Meanwhile, many studies have identified that C4 might be a biomarker for forecasting systemic lupus erythematosus [33–34]. Therefore, C4 might be related to acute inflammation and the immune response. Additionally, serum C4 level was also correlated with atherosclerosis. In 1988, Muscari et al. [35] studied the relationship between humoral immunity and atherosclerosis, and the results indicated that the C4 level might be an independent factor associated with atherosclerosis. Shields et al. [36] investigated the complement system, vascular stiffness, and atherosclerosis, and suggested that C4 might play a role in vascular stiffness and atherosclerosis. Considering the function of C4, we had reason to believe that the C4 level was associated with development of cardiovascular disease. To some extent, serum HCY might also be correlated with C4 concentration in inducing the development and progression of diseases.

In the linear regression analysis, CRP was also discovered to be associated with HCY level [unadjusted: *Beta* = 0.056, 95%*CI* = (0.037, 0.740), *P* = 0.030]. The positive correlation between the HCY level and CRP has been confirmed in many other studies. In 2006, Holven et al. [20] showed that the serum concentration of CRP was significantly elevated among hyperhomocysteinemic subjects. As one of the bio-humoral parameters of inflammation, the importance of CRP should not be ignored. In order to investigate more potential correlations, an additional multivariate logistic regression analysis was performed. In this analysis, the results not only confirmed a significant association between the HCY level and C4, but also identified a new relationship within IgM and HCY [unadjusted: *OR* = 1.427, 95%*CI* = (1.052, 1.936); age-adjusted: *OR* = 1.446, 95%*CI* = (1.061, 1.970); multivariate adjusted: *OR* = 1.447, 95%*CI* = (1.062, 1.973)].

IgM is produced by B cells and is one of the immunoglobulins that take part in the process of humoral immune defense. It is the first antibody that appears in the initial immune reaction. And the increasing concentration of IgM indicated a recent infection [37]. However, studies about the association between the HCY and IgM levels are limited. Muscari et al. conducted a study about humoral immunity in diffuse atherosclerosis, but did not identify any significant correlation between IgM and atherosclerosis [35]. Even so, in 2008, Kerekes et al. [38] suggested that HCY and IgM might be involved in the development of vascular disease in rheumatoid arthritis. Above all, the association between IgM and HCY remained unclear. According to our results, we also suspected that IgM and HCY might interact through some potential mechanisms.

Although researches about the mechanism of HCY influencing the immune system was limited recently, the correlations between HCY and immune cells including T and B lymphocytes were explicit. In 2004, Dawson et al. [39] suggested that HCY could activate T lymphocytes, thus inducing cytokine secretion. In addition, Chang et al. [40] proposed that HCY could regulate B lymphocytes, thus increasing IgG production via the ROS-NF-kappaB pathway. However, the potential mechanism of HCY regulating the other immune factors, such as C4, CRP, and IgM was unclear. Considering the association between HCY and lymphocytes, we speculate that HCY might influence the immune factors discovered in our studies through some potential pathways. The further studies might be mainly focused on the aspects of genetic regulation, pathway analysis and protein expression, which would help us understand the functions of HCY in the immune system more clearly.

### Limitations

After a comprehensive analysis, we discovered that C4, CRP, and IgM might be significantly associated with HCY levels. However, there were some limitations that should not be ignored: (I) this study was a cross-sectional analysis that reflected the status of a population in a particular period. Therefore, there might be some bias in the results and conclusions. (II) This study was based on the available data in the *FAMHES* project. There were only six inflammatory/immune items included, which may not reflect the overall conditions of the inflammatory/immune system. (III) Studies about the relationship between C4, CRP, IgM, and HCY levels are limited. The mechanisms and interactions are unclear.

## Conclusions

HCY is an amino acid with various functions that takes part in diverse functions related to many diseases. HCY has been said to be associated with the inflammatory/immune system. Our results suggest that C4, CRP, and IgM in serum are significantly associated with HCY concentration. However, further studies about the mechanism of the interaction are needed.

## Supporting Information

S1 Table The primary data used in the study for C3. Drink
A01: 1 drinking; 2 no drinking.
Smoke
B01: 1 smoke; 2 no smoke.
BMI = weight / (height/100)^2^, WHR = waist/buttocks.</SI_Caption>
(XLS)

S2 Table The primary data used in the study for C4. Drink
A01: 1 drinking; 2 no drinking.
Smoke
B01: 1 smoke; 2 no smoke.
BMI = weight / (height/100)^2^, WHR = waist/buttocks.</SI_Caption>
(XLS)

S3 Table The primary data used in the study for CRP. Drink
A01: 1 drinking; 2 no drinking.
Smoke
B01: 1 smoke; 2 no smoke.
BMI = weight / (height/100)^2^, WHR = waist/buttocks.</SI_Caption>
(XLS)

S4 Table The primary data used in the study for IgA. Drink
A01: 1 drinking; 2 no drinking.
Smoke
B01: 1 smoke; 2 no smoke.
BMI = weight / (height/100)^2^, WHR = waist/buttocks.</SI_Caption>
(XLS)

S5 Table The primary data used in the study for IgG. Drink
A01: 1 drinking; 2 no drinking.
Smoke
B01: 1 smoke; 2 no smoke.
BMI = weight / (height/100)^2^, WHR = waist/buttocks </SI_Caption>
(XLS)

S6 Table The primary data used in the study for IgM. Drink
A01: 1 drinking; 2 no drinking.
Smoke
B01: 1 smoke; 2 no smoke.
BMI = weight / (height/100)^2^, WHR = waist/buttocks. </SI_Caption>
(XLS)
